# Lessons from a Rubik's Cube to solve the biodiversity crisis

**DOI:** 10.1111/cobi.14416

**Published:** 2024-11-19

**Authors:** Ana M. M. Sequeira, Erika J. E. Techera

**Affiliations:** ^1^ Division of Ecology and Evolution, Research School of Biology The Australian National University Canberra Australian Capital Territory Australia; ^2^ UWA Oceans Institute and School of Biological Sciences The University of Western Australia Perth Western Australia Australia; ^3^ UWA Law School and UWA Oceans Institute The University of Western Australia Perth Western Australia Australia

**Keywords:** global biodiversity framework, marine megafauna conservation, science‐policy interface, socioecological boundary, sociopolitico‐legal overlap, conservación de la megafauna marina, frontera socio‐ecológica, interfaz científico‐política, marco mundial de biodiversidad, traslape sociopolítico‐legal

## Abstract

Global biodiversity is facing unprecedented pressures, calling into question the effectiveness of existing governance systems aimed at halting extinctions. Renewed hope arose with the recent Conference of the Parties (COP) to the Convention on Biological Diversity (COP15 December 2022) and the Convention on International Trade in Endangered Species (COP19 November 2022). Yet, barriers remain that hamper biodiversity conservation. Identifying and overcoming these barriers is crucial for success. We considered previous lessons learned to show that current barriers to conservation are centered on a multidimensional array of mismatches among legal (law), ecological (science), and sociocultural (human) dimensions across the short, medium, and long term. Focusing on highly migratory marine megafauna (whales, sharks, and turtles), we used the Rubik's cube as a metaphor to conceptualize the multidimensional mismatches and devised a pathway for solutions that is highly dependent on strict alignment across all dimensions. We recommend the continuous cycling across all dimension interfaces to align the use (and update) of regulations and processes in law, improve data and experimentation methods in science, and develop education and engagement actions in the human dimension. This timely alignment across all dimensions is key to achieving biodiversity targets and avoiding further extinctions.

## Abstract (es)

Lecciones del cubo Rubik para solucionar la crisis de biodiversidad

**Resumen:**

La biodiversidad mundial enfrenta una presión sin precedentes, lo que cuestiona la efectividad de los sistemas de gestión dirigidos a detener las extinciones. En la reciente Conferencia de las Partes (COP) ante el Convenio sobre la Diversidad Biológica (COP15, diciembre 2022) y la Convención sobre el Comercio Internacional de Especies Amenazadas (COP19, noviembre 2022) surgió una esperanza renovada; sin embargo, todavía existen barreras que dificultan la conservación de la biodiversidad. Es importante identificar y sobrepasar estas barreras para tener éxito. Consideramos los aprendizajes previos que muestran que las barreras actuales para la conservación están centradas en una colección multidimensional de desfases entre las dimensiones legal (ley), ecológica (ciencia) y sociocultural (humana) a corto, mediano y largo plazo. Nos enfocamos en la megafauna marina con alta migración (ballenas, tiburones y tortugas) y usamos el cubo Rubik como metáfora para conceptualizar los desfases multidimensionales y diseñar una vía para soluciones que sea en gran parte dependiente de la alineación estricta en todas las dimensiones. Recomendamos un ciclo continuo en todas las interfaces de las dimensiones para alinear el uso (y actualización) de las regulaciones y procesos de la ley, mejorar los métodos de experimentación y la información en la ciencia y desarrollar acciones educativas y participativas en la dimensión humana. Esta alineación oportuna de todas las dimensiones es clave para que se alcancen los objetivos de conservación de la biodiversidad y se eviten más extinciones.

## INTRODUCTION

**FIGURE 1:**
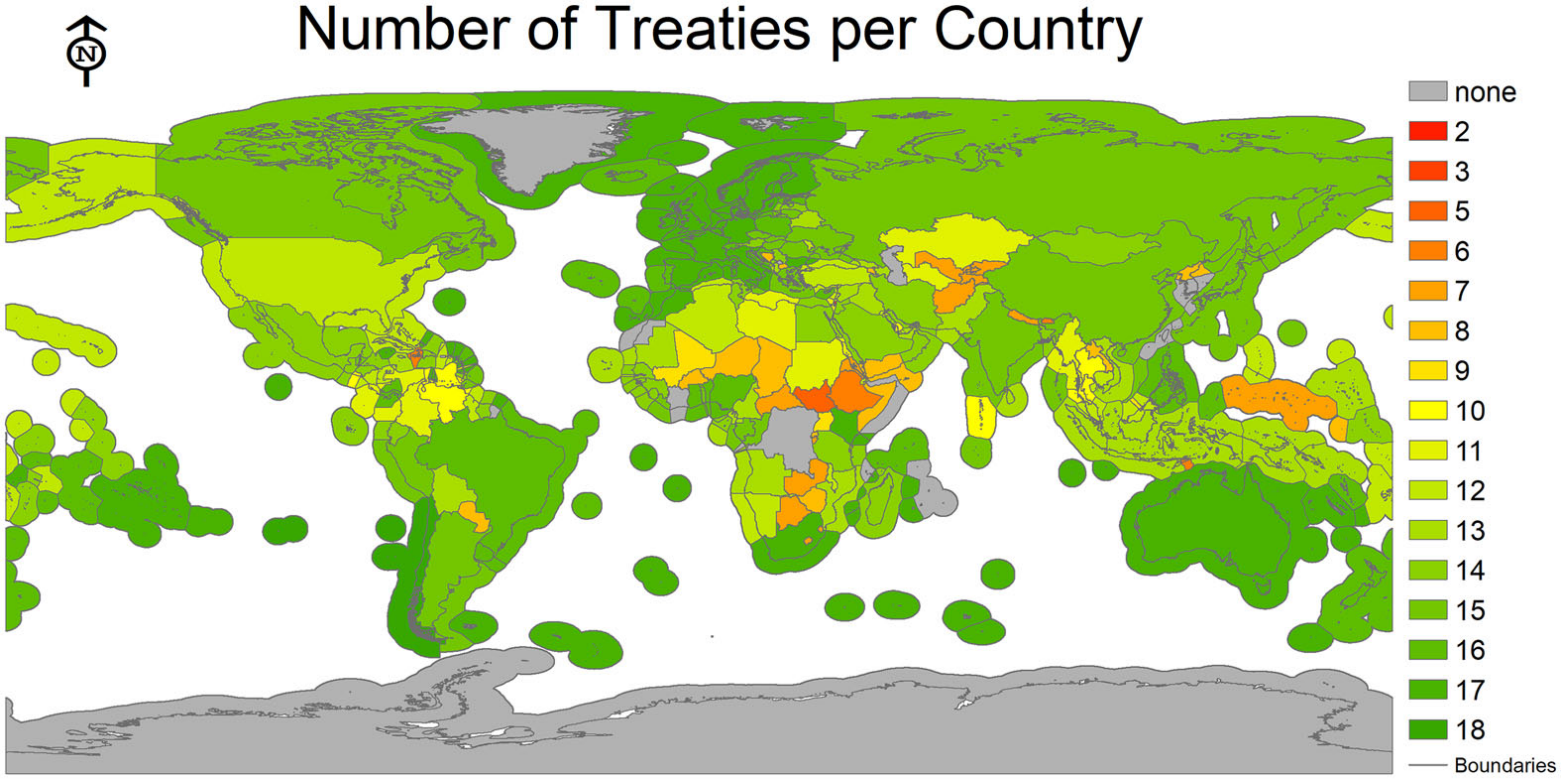
Number of international legal instruments (treaties, protocols, and annexes) adopted by each United Nations’ state party sovereign territory (including territorial land, seas, and exclusive economic zones) (treaties.un.org/Pages/AdvanceSearch.aspx?tab = UNTS&clang = _en) relevant to the marine environment to which each state is a party.

Accelerated biodiversity loss due to anthropogenic pressure is leading to the sixth mass extinction: the Anthropocene extinction (Barnosky et al., 2011; Ceballos et al., 2020; Cowie et al., 2022; Pievani, 2014; Wake & Vredenburg, 2008). In particular, megafauna are 3 times more threatened with extinction than most other vertebrate taxa (Ripple et al., 2019). This includes about one‐third of marine megafauna species (Estes et al., 2016) across elasmobranchs (Dulvy et al., 2021), marine mammals (Avila et al., 2018), and seabirds (Dias et al., 2019). Despite observed population declines (iucnredlist.org; Estes et al., 2011; McCauley et al., 2015), rebuilding marine life has been highlighted as a “doable grand challenge for humanity” (Duarte et al., 2020). Indeed, halting human‐induced extinctions of threatened species is goal 1 of the recently adopted Kunming‐Montreal Global Biodiversity Framework (GBF) (15th Conference of Parties of the UN Convention on Biological Diversity [CBD] December 2022). However, despite multiple international environmental agreements ratified by the majority of relevant countries (Table 1 & Figure 1), the failure to achieve previously set targets (e.g., Aichi Targets) calls into question their effectiveness and ability to achieve biodiversity conservation goals. Understanding the barriers that hinder conservation is, therefore, crucial for the timely recovery of marine populations. Lessons from known success stories can shed light on the complexity of issues that need to be addressed and provide ideas to advance biodiversity conservation.

**TABLE 1:** List of global environmental law frameworks adopted by United Nations Member States relevant to biodiversity conservation^*^.

Acronym or name		Definition	Parties
BBNJ Agreement	Biodiversity Beyond National Jurisdiction, also known as High Seas Treaty.	international agreement under the United Nations Convention on the Law of the Sea focused on the conservation and sustainable use of living marine biodiversity in areas beyond national jurisdiction through high seas marine protected areas, environmental impact assessment, capacity building, and the regulation of marine genetic resources	8 with 86 further signatories
CBD (cbd.int)	Convention on Biological Diversity	global agreement providing a framework for the conservation and sustainable use of biological diversity and its components, including obligations for state parties to develop national biodiversity strategies and action plans	194
CITES (cites.org)	Convention on International Trade in Endangered Species of Wild Fauna and Flora	multilateral treaty to protect endangered plants and animals by regulating the threats of international trade	183
CMS (cms.int)	Convention on the Conservation of Migratory Species of Wild Animals	international agreement that aims to list and conserve migratory species by engaging all state parties through which a listed species range	133
ICRW (iwc.int)	International Convention for the Regulation of Whaling	created to provide for the proper conservation of whale stocks and thus make possible the orderly development of the whaling industry	88
London Conv (imo.org/en/OurWork/Environment/Pages/London‐Convention‐Protocol.aspx)	London Convention—Convention on the Prevention of Marine Pollution by Dumping of Wastes and Other Matter	one of the first global conventions to protect the marine environment by regulating the deliberate dumping and disposal of waste; The London Protocol later adopted to strengthen the London Convention by prohibiting the dumping of all waste with limited exceptions	87
MARPOL (imo.org/en/OurWork/Environment/Pages/Pollution‐Prevention.aspx)	International Convention for the Prevention of Pollution from Ships	developed by the International Maritime Organization to minimize pollution of the oceans and seas through incidental and accidental release of pollutants; has 6 annexes: Annex I: Prevention of Pollution by Oil; Annex II: Control of Pollution by Noxious Liquid Substances in Bulk; Annex III: Prevention of Pollution by Harmful Substances carried by Sea in Packaged Form; Annex IV: Prevention of Pollution by Sewage from Ship; Annex V: Prevention of Pollution by Garbage from Ships; Annex VI: Prevention of Air Pollution from Ships	161
PARIS AGMT (unfccc.int/process‐and‐meetings/the‐paris‐agreement)	Paris Agreement	international treaty on climate change that covers climate change mitigation and adaptation by requiring parties to submit nationally determined contribution documents	193
RAMSAR (ramsar.org)	Ramsar Agreement on Wetlands of International Importance	international treaty for protection of wetland habitats requiring state parties to list at least 1 wetland and provide law and policy for its wise use	171
UNCLOS (un.org/depts/los/convention_agreements/convention_overview_convention.htm)	United Nations Convention on the Law of the Sea	international legal agreement setting forth rights, duties, and obligations of states, establishing maritime jurisdictional zones and maritime activities, including optimum use, conservation, and management of living marine resources and protection and preservation of the marine environment	168
UNFSA (un.org/depts/los/convention_agreements/convention_overview_fish_stocks.htm)	United Nations Fish Stocks Agreement	conservation and management of straddling and highly migratory fish stocks	92
UNFCCC (unfccc.int/process‐and‐meetings/what‐is‐the‐united‐nations‐framework‐convention‐on‐climate‐change)	United Nations Framework Convention on Climate Change	international environmental treaty to combat “dangerous human interference with the climate system,” in part by stabilizing greenhouse gas concentrations in the atmosphere	197
WHC (whc.unesco.org/en/convention)	World Heritage Convention	international treaty for the conservation and management of listed natural and cultural heritage sites with outstanding universal value	196

* Information presented drawn from data in the UN Treaty Collection (treaties.un.org/Pages/AdvanceSearch.aspx?tab = UNTS&clang = _en; accessed September 2022).

Regulation of whaling is an excellent example of a success story from which lessons can be learned; it has led to the rebound of some whale populations that were on the brink of extinction in the mid‐1950s (Bejder et al., 2016). Historically, whales were hunted mostly for their oil (McVay, 1966), but harvesting continued even after the development of alternatives (e.g., fossil fuels) (York, 2017). The International Convention on the Regulation of Whaling (ICRW), which is still in force today, was adopted in 1946, established the International Whaling Commission (IWC), and was focused on preventing the overharvesting of whales. However, the regulations adopted were loose, with high catch quotas, and whale numbers continued to decline. By the 1970s, environmentalism had become a major concern, with the United States championing conservation laws and including several whale species on its endangered species list. The timely release of the album *Songs of the Humpback Whale* (Payne & Mcvay, 1971) increased public support for whale conservation. At the same time, as substitutes for whale products became more common and whale numbers continued to decline, the whaling industry became more costly. In 1982, the IWC called for a global moratorium on commercial whaling, which was ultimately successful in 1986 and led to a transition from whale hunting to whale conservation. The successful conservation of whales is, therefore, a result of the synergistic alignment of 3 important components: growing scientific evidence for whale population declines and technological developments leading to replacement products not derived from whales, the existence of an international agreement and institution that was able to flexibly respond to the challenge and facilitate whale protection and conservation, and a shift in human values reflected in public support for whale conservation combined with declining socioeconomic returns from whaling. We argue that these 3 components need to align within a similar period to facilitate an optimum response to scientific knowledge and lead to effective conservation.

The whale example highlights the multidimensional complexity of biodiversity conservation (particularly for marine megafauna) that clearly hinges on the strict alignment across the science (ecology), law (legal), and the human (sociocultural) interfaces within specific time frames. Other similarly synergistic alignments have also benefitted species conservation. For example, fish populations rebounded (scientific knowledge) during World War II (social context) following the forced closures (emulated legal measures) for fishing in the North Sea due to human conflict (Beare et al., 2010). When the war ended (social context), fishing resumed (implicit scientific knowledge of populations rebounding), and in the Atlantic, the Cod War was so severe that the international community had to negotiate a new global agreement, resulting in the United Nations Convention on the Law of the Sea (UNCLOS, 1982) (legal outcome). Emerging scientific evidence also demonstrates a coincidental reduction in impacts on biodiversity during the COVID‐19 pandemic (social context) (Bates et al., 2020; Lecchini et al., 2021). Examples include the reduction of ships traffic lowering noise pollution that affects whales (Dunn et al., 2021; Ryan et al., 2021). After travel restrictions were eased (legal enforcement), ship traffic resumed, and impacts continued (sociocultural element). These examples highlight the importance of the human component in changing activities that may be harmful for biodiversity conservation. We argue that the focus of scholarship to date has been on the science‐law interface (much scholarship refers to policy, but our focus here is law [Cosens et al., 2017]), without due attention to the tridimensionality involving the Human dimension, which is critical for successful biodiversity conservation.

**FIGURE 2:**
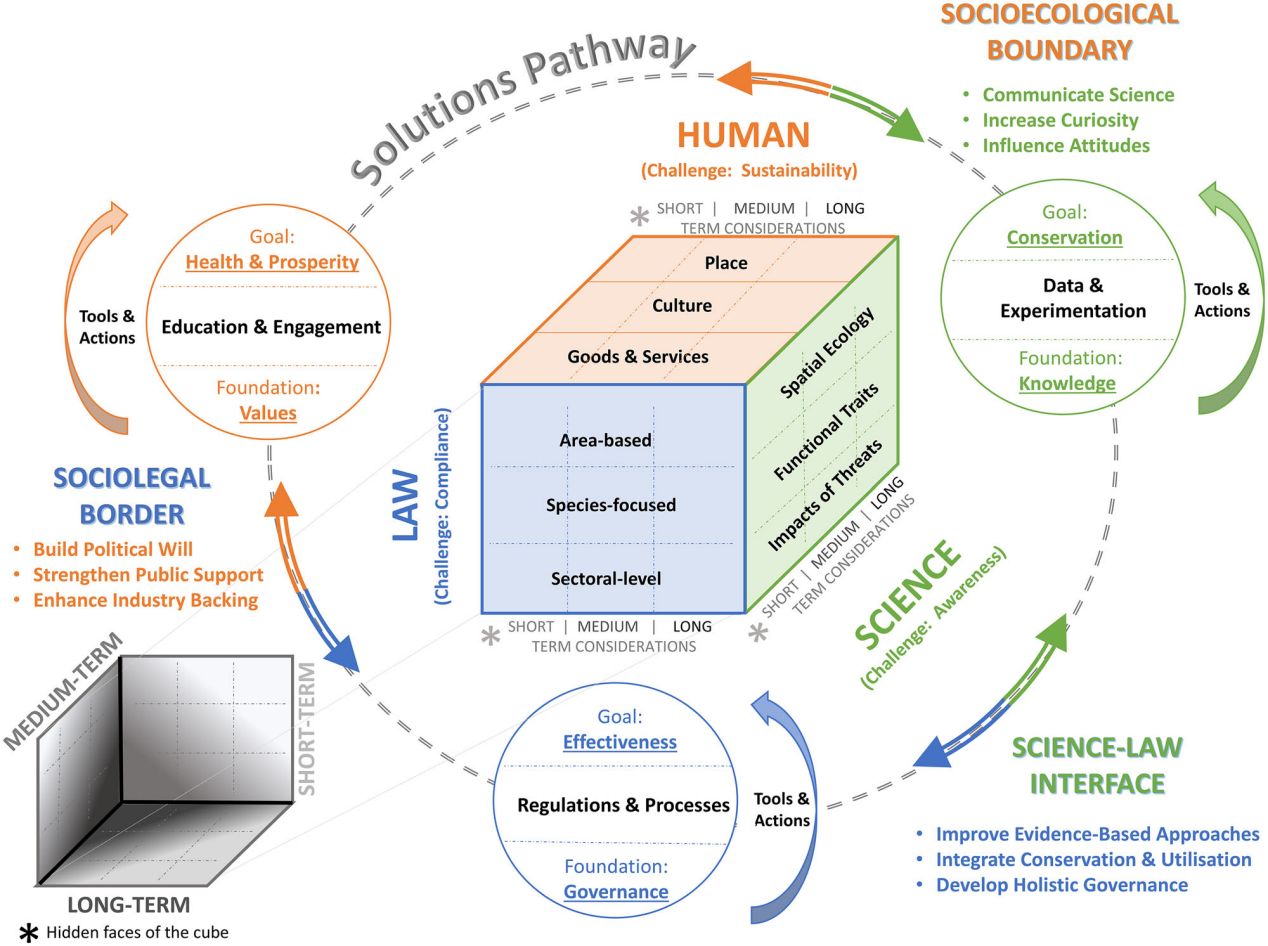
Rubik's cube solution to biodiversity conservation (center cube, multidimensional aspect of mismatches across the law, science, and human dimensions; hidden faces of the cube on the bottom left, temporal dimensions across the short, medium, and long term).

We expanded on the various elements of science, law, and human dimensions and particularly on the necessity for alignment at their interfaces across 3 temporal dimensions: short, medium, and long term (Figure 2). Conceptualizing such multidimensional complexities can be likened to a Rubik's Cube, where alignments across each face are needed if the problem is to be resolved (similar to Trivedy, 2020). The successful implementation of current global biodiversity frameworks and the achievement of conservation targets are, therefore, contingent on how the existing mismatches across any of these interfaces are addressed (Table 2). Although we focused on the needed alignment to address existing mismatches, some matches also occur, and are known to lead to successes (see Falco et al., 2022).

**TABLE 2:** Examples of existing mismatches^*^22 across interfaces of the law, science, and human dimensions.

Dimension	Example mismatch	Reference
Science		
Spatial Ecology	Species ranges and their migratory behavior do not match geopolitical boundaries. Areas of high ecological importance are not necessarily protected or assessed based on single species or functional uniqueness. Regions of high biodiversity often match locations where people live or work (i.e., where industry takes place), resulting in conflicting values.	Beal et al. (2021); Conners et al. (2022); Harrison et al. (2018)
species or functional traits	Entire species’ life cycles are not considered by area‐based regulations. Functional role may be disregarded in species‐focused regulations. Species life‐history traits are often not considered in sectoral‐level regulations.	Conners et al. (2022); Hays et al. (2014)
impacts from threats	Impacts from industry affect species across multiple areas at various spatial and temporal scales. Some of the most affected species also have the most unique functional roles and are not protected. Priorities are given to goods produced, services provided, and sociocultural values over species impacts and conservation.	Fossette et al. (2014); Queiroz et al. (2019); Sequeira et al. (2019); Womersley et al. (2022)
Law		
area based	Protected areas do not match the places or times that species need (i.e., there are geographical and temporal mismatches). Protected areas are not defined by species’ traits or places most closely linked to human impacts and industries. Area‐based approaches may not respond effectively to human impacts and industries (reflecting poor choice of legal tool).	Conners et al. (2022); Lindegren et al. (2018)
Species Focused	Species‐based approaches do not include all areas, species, or industries associated with the impacts. Species‐based approaches do not account for species’ area‐based needs at different stages of the life cycle. Protected species are not necessarily aligned with culturally significant species or those most affected.	Frank and Wilcove (2019); Sky (2010)
sectoral level	Sectoral approaches do not account for multiple industries that may affect the same species. Sectoral regulations do not account for spillover effects where industry may be excluded. Sectoral approaches often exclude operations within protected areas.	Attard et al. (2024); Pirotta et al. (2019)
Human		
Place	Places where people live or work may not align with goods and services produced there. Places of cultural importance often match areas of high ecological significance. Places where significant goods and services are produced are often those where species need protection.	Berkes (2012); Verschuuren et al. (2010)
Culture	Multiple sociocultural norms and values may be exhibited within one place. Sociocultural norms and values may not align with goods and services produced or ecological significance of a place. Protected areas do not align with (or accommodate) sociocultural norms and values.	Vierros et al. (2020)
goods and services	Goods and services may be produced in a place to support other places, cultures, and people. Production and extraction that affect species may occur in protected areas and provide jobs or food security. Goods and services may align with sectoral regulations but not with sociocultural needs.	Salgueiro‐Otero and Ojea (2020)

* We present examples across the main 3 dimensions of the Rubik's cube represented in the center of Figure 2. In these examples, we divided each dimension into site‐specific issues (place, area, spatial ecology), concerns intrinsic to each dimension (human culture, focused regulations and traits of species), and use‐related aspects (focusing on goods and services, sectoral actors, and impacts on species). Each aspect needs to align in time for successful biodiversity conservation. References include some that apply across dimensions, and the “example mismatch” reflects a perspective consistent with the dimension being highlighted.

## MISMATCHES AT THE SCIENCE‐LAW INTERFACE

The majority of scholarship on existing mismatches focuses on the science‐law interface, which is listed as a cause of ineffectiveness that can lead to failure to achieve conservation goals for biodiversity (Crowder et al., 2006; Falco et al., 2022). For marine megafauna, such mismatches mostly focus on spatial incongruities between existing marine protected areas (MPAs) and known areas of importance to marine megafauna (Conners et al., 2022), temporal mismatches associated with reactive (rather than proactive) legal processes (e.g., in listing species on appendices to the Convention on International Trade in Endangered Species of Wild Fauna and Flora [CITES, 1973]), and ecological value mismatches relating mostly to the lack of legal considerations for species functional traits and values. These mismatches have been reported in specific regions (Brooks et al., 2016; Harrison et al., 2018; von Heland et al., 2014) and jurisdictions (Crowder et al., 2006; Lagabrielle et al., 2018) in relation to particular species (Havice et al., 2018) or aspects of conservation (e.g., use of data for policy; Lindegren et al., 2018) and tend to concern single conservation tools (e.g., MPAs) (Maina et al., 2020) or highlight the focus on agreement making rather than implementation (Beal et al., 2021).

Most of these mismatches stem from the focus on human rather than species’ needs in different nations, and we focused on relevant international law regimes because marine megafauna travel across jurisdictions. Therefore, common and collaborative arrangements, as captured in international treaties and regional organizations, can play a harmonizing role based on their ability to garner common interests and shared commitments across states. However, international law regimes are usually divided into regulations on natural resources use (e.g., fisheries regulations), nonextractive industry measures (e.g., shipping laws), and environmental agreements (e.g., Convention on Migratory Species; CMS, 2017). These legal regimes are inherently linked to sovereignty and politico‐legal boundaries, such as existing jurisdictions and their exclusive economic zones (Figure 1), which are not aligned with species movements. In the high seas (areas beyond national jurisdiction), living marine resource management occurs mostly through regional fisheries management organizations (RFMOs) that focus on extractive industry‐based operations (e.g., tuna fisheries) and rely on member states enforcing laws against their own vessels and nationals. Beyond the Convention on Migratory Species (CMS), there is no specific international law, environmental agreement, or collaborative intergovernmental organization responsible for marine megafauna conservation globally. The recent adoption of the Biodiversity Beyond National Jurisdictions (BBNJ) Agreement (https://tinyurl.com/3vztz8kv) under the United Nations Convention on the Law of the Sea (UNCLOS) provides the potential for more holistic and integrated conservation measures in the high seas (Kim, 2024; Santos et al., 2022). Furthermore, in its recent advisory opinion (case 31, 21 May, 2024), the International Tribunal on the Law of the Sea (ITLOS) confirmed that UNCLOS is not to be read as a static instrument; rather, it is to be interpreted in light of State obligations in later instruments (Yiallourides & Deva 24 May, 2024). Both developments may play a harmonizing role that will enhance ocean governance and may assist marine megafauna conservation.

## CHALLENGES OF CONSERVING MARINE MEGAFAUNA

Despite the existing legal interventions and the key roles marine megafauna play in maintaining the health of marine ecosystems (Estes et al., 2016), many species are now at high risk of extinction (Avila et al., 2018; Dias et al., 2019; Dulvy et al., 2021; Estes et al., 2011; Ripple et al., 2019) (also see iucnredlist.org for conservation statuses listed for species). Conservation of marine megafauna is challenging for many reasons (including some lack of data), yet we argue that the following 3 reasons are paramount (Figure 2, central cube). First, marine megafauna spatial ecology is widespread including global distributions (e.g., Sequeira et al., 2014). Many animals cross multiple politico‐legal boundaries (Beal et al., 2021; Harrison et al., 2018; Vierros et al., 2020) and thus connect and influence distant ecosystems (McCauley et al., 2012; Roman et al., 2014) that feature in several legal frameworks. Second, their species‐specific traits are widely variable, including species that are air or gill breathers with different migratory and diving behaviors (Andrzejaczek et al., 2020; Braun et al., 2022; Costa & Favilla, 2023), different life‐history traits (e.g., Cortés, 2000), are cold or warm‐bodied (Grady et al., 2019), and nest or rest on coastal shores (Palomino‐González et al., 2020). Third, estimating the cumulative risks and impacts of anthropogenic threats is difficult because existing threats (Halpern et al., 2008; Sequeira et al., 2019), which fall under diverse legal regimes, affect species differently depending on their taxon and life‐cycle phases.

To restore marine megafauna stocks (Duarte et al., 2020) and avoid extinctions similar to those already observed for terrestrial megafauna (Ripple et al., 2019), there is an immediate need to focus on achieving biodiversity targets (e.g., GBF). We argue that these can be achieved through better integration of scientific ecological data into existing legal frameworks (industry‐related and environmental regimes) and more efficiency in mitigating cumulative impacts from anthropogenic threats to species. Law has a powerful role to play in this space with international treaties setting global standards (Table 1), creating binding obligations for member states, and providing forums for sharing knowledge and solutions (see Figure 1 for number of treaties ratified per country). Nevertheless, the difficulties of responding to the 3 challenges above are exacerbated by weaknesses in the law, including fragmentation across legal regimes, differences in domestic implementation of international obligations, and inherent problems with compliance and enforcement. Improving the effectiveness of existing international law regimes is, therefore, of paramount importance. The 2024 ITLOS Advisory Opinion is likely to significantly improve fragmentation because it confirmed State obligations at the intersection of climate change, environment, and oceans governance, including the requirement for stringent due diligence informed by the best available science (Yiallourides & Deva 24 May, 2024). The BBNJ Agreement offers the potential to improve effectiveness including through novel voting provisions, rather than relying on consensus decisions, which may avoid some states blocking conservation measures (Liu, 2024). Nevertheless, although the BBNJ Agreement has received many State signatories, it may still take some years to come into force (Blasiak & Jouffray, 2024). It is vital, therefore, to find ways to encourage State ratification to speed up this process.

## INTERNATIONAL LAW REGIMES AND THEIR LIMITATIONS

Existing global frameworks usually use 1 (or more) of 3 types of legal tools (Figure 2, central cube). One type is area‐based approaches, such as those that feature as targets in the recently adopted Global Biodiversity Framework (i.e., 30% area protection by 2030). Area‐based approaches are often implemented through the creation of MPAs (which are then included in shipping and fishing regulations), but these are frequently designated where the potential for user conflict is anticipated to be low (Conners et al., 2022; Lindegren et al., 2018) rather than based purely on different species needs. Another tool utilized is species‐focused provisions that protect marine biodiversity through (hierarchical) species listings based on scientific evidence, such as CITES and CMS. Species‐focused provisions are not comprehensive. They tend to prioritize extraction over conservation for species with current high commercial value (e.g., tuna and sharks) and to favor species that have been commercially harvested or have well‐endorsed conservation value (e.g., almost all whales subjected to International Whaling Commission management and all seven species of marine turtles are included in CITES). Where megafauna species are listed, it is often after some member states use political and procedural tactics that delay timely conservation interventions (e.g., history of shark listings under CITES) (Sky, 2010). Delays in species listings may mean that human impacts, such as international trade, continue long after their risk species extinctions is known (Frank & Wilcove, 2019). A final type of approach is industry‐related measures, which regulate the industry or activity that directly impacts on species, such as commercial fishing (e.g., via RFMOs). Industry‐related measures inherently deal with competing interests between species needs and human requirements (i.e., livelihoods and the production of goods and services), yet their mandates are squarely focused on the utilization of stocks to maintain the industry and that often leads to compromises favoring human activities (e.g., the agreement for the establishment of the Indian Ocean Tuna Commission [Secretary of State for Foreign & Commonwealth Affairs, 2019, Art V]).

Despite the wealth of environmental laws, policies, and institutions across all governance scales (see Table 1 & Figure 1), international frameworks ultimately rely on political will. When states become a party to treaties, they signal an agreement with the underlying purpose of the instrument and a willingness to adhere to the international obligations. But treaties frequently include voting mechanisms that allow states to veto or block listings or to negotiate weak outcomes even when scientific evidence is clear on the conservation needs. States are also not legally obliged to become parties to a global legal instrument and may withdraw from it at any time. For example, Japan withdrew from the ICRW in 2019 to resume commercial whaling despite significant international pressure (Kolmas, 2021). Indeed, despite the success observed in the rebound of whales from targeted hunting for oil, many whale species continue to be threatened with extinction due to other anthropogenic threats that include fishing, shipping, and climate change (Attard et al., 2024; Pirotta et al., 2019). Member State implementation and compliance is critical to the success of any legal regime and is, in turn, linked to human priorities and stakeholder engagement.

## INFLUENCE OF THE HUMAN DIMENSION ON EXISTING MISMATCHES

Incorporating human dimensions in conservation interventions improves wildlife outcomes (Bennett et al., 2017; Serota et al., 2023), yet there are limited studies on interactions between social‐ecological systems (Salgueiro‐Otero & Ojea, 2020) and the role of law (Ebbesson & Hey, 2013). Public opinion and industry lobbying influence political will to adopt, implement, and enforce legal frameworks. Compliance with adopted laws is critical to their success and depends on public support and private industry endorsement, which are directly linked with economic drivers and sociocultural motivations for noncompliance (Oyanedel et al., 2020). All of these aspects make up the human dimension from which we drew out 3 major components (Figure 2, center cube). The first relates to places where people live or work, where relevant industrial activities occur, or areas of particular significance to communities (religious or otherwise). The second relates to culture (including political, social, and cultural elements) and is based on intrinsic ethos, representing philosophies, norms, beliefs and values, attitudes and customs, or generational knowledge. The third is based on the production and consumption of goods and services, which is the foundation of economies, jobs, and security. Whereas human activities are a major driver of environmental degradation, people and societies rely on nature for sociocultural benefits (Salgueiro‐Otero & Ojea, 2020). These components, therefore, play a key part in the effectiveness of conservation efforts and legal frameworks, meaning the human dimension is a driving force for the alignment of existing mismatches with science and law (Figure 2, and see Table 2 for examples of existing mismatches at the interplay of the 3 dimensions).

Mismatches at the intersection of humans and science (the socioecological boundary) can be addressed with increased awareness and knowledge exchange, for which education and science communication is essential (Contera, 2021) to raise curiosity, increase connection, and influence attitudes and decision‐making (Burns et al., 2003; Karcher et al., 2021). Mismatches at the intersection of humans and law (the sociopolitico‐legal overlap) can similarly be addressed through improved education and by taking a participatory and collaborative (rather than top‐down) approach to law making. This will ensure alignment with (and facilitate changes in) sociocultural values, which ultimately drive public support and political will (Winter & May, 2001). Where laws align with norms and values, and provide little or no disruption to goods and services, compliance is more likely (Winter & May, 2001). Although the human dimension includes a myriad of aspects that we could not explore here, there is a need to identify how best to implement international law in different national contexts, including ways to harness motivations to comply, which ultimately will lead to successful outcomes (Wagner et al., 2020). Politico‐legal tensions and socioeconomic motivations, which are often a reflection of a mismatch of human values, complicate the development of new laws and implementation of regulations for marine megafauna conservation (e.g., Sowman & Sunde, 2018). Therefore, decision‐makers must genuinely engage with stakeholders (i.e., the general public, civil society groups, and private industry) to ensure sociocultural and economic needs are met.

## TEMPORAL COMPONENTS AND NEED FOR ALIGNMENT ACROSS MISMATCHES

Identifying and addressing mismatches beyond the science‐law interface and the simultaneous alignment across the science, law, and human dimensions in the short, medium, and long term (Figure 2—hidden faces of the Rubik's cube) will lead to the greatest positive outcomes for biodiversity conservation. There are a large number of potential temporal mismatches that we could not cover here, but we provide some examples.

For many mismatches, scientific evidence has revealed successful outcomes from technological advances that are ready to be implemented (short term), as is the case in fisheries, where adaptation of fishing gear helps avoid bycatch or reduces marine megafauna entanglement (Allman et al., 2021; Guidino et al., 2022). There is also substantial scientific evidence for mismatches between areas protected and important sites for some species (Conners et al., 2022; Lindegren et al., 2018). With the recent decision to protect 30% of the world's oceans by 2030 (a medium‐term goal), a scientific, holistic approach to identify and map biologically and ecologically important areas across marine megafauna taxa at global scale is needed to better inform the implementation of targeted protection areas. For this process, it is important to consider the spatial, temporal, and ecological relevance of species and to specifically incorporate the extensive work already done in this regard through the delineation process to identify important areas, such as important marine mammal areas (Hoyt & Notarbartolo Di Sciara, 2021) and important shark and ray areas (Jabado et al., 2023). In the long term (e.g., later in the century), some species might shift their distributions due to climate change, and the alignment of protected areas will need to take those changes into consideration (e.g., Buenafe et al., 2023).

Previously, we mentioned temporal mismatches between the science and law dimensions, for which alignment requires greater understanding of the role of science in decision‐making (van Kerkhoff & Pilbeam, 2017). Particularly, the timing of decisions to enhance protection for certain species is critical, as delays in listing species under environmental law regimes (e.g., CITES) when scientific data have demonstrated evidence for damaging impacts of international trade in those species allows the impacts to continue (Frank & Wilcove, 2019). This temporal mismatch is contrary to the aims of environmental law regimes, which make explicit reference to the scientific value of wildlife (CMS Preamble; CITES Preamble) and decisions based on scientific evidence and data (CMS Article III; CITES Art XV). Therefore, temporal mismatches between scientific knowledge and legal action undermine the objectives of these regimes. Temporal mismatches between the acquisition of scientific knowledge and legal action can be attributed, in part, to a lack of political will and public support to respond. This demonstrates the importance of the human dimension and sociocultural values that are based on knowledge as well as vested beliefs and interests (Pascual et al., 2023). Both public perception and political will are informed by knowledge of scientific findings that may lag behind academic publication of new data due to the complexity of science communication (Meenakshi, 2021; NASEM, 2017). Yet, despite a demonstrated need for transformative change in human behavior, shifting sociocultural values takes longer time scales (Pascual et al., 2023). Similarly, a mismatch between the human and law dimensions may extend the time for implementation of, and compliance with, new legal regimes. The form of engagement and timing of public participation in law reform and decision‐making are important factors (Chess & Purcell, 1999). In addition, existing laws tend to support sociocultural interests in environmental resources (e.g., property rights) and present a counterproductive barrier to the uptake of more nature‐centered values (Pascual et al., 2023).

## THE SOLUTIONS PATHWAY

To overcome mismatches, tools and actions must be developed and maintained at each dimension, including the use and update of regulations and processes for law, data and experimentation for science, and education and engagement for the human dimension. Such tools and actions are key to advancing each dimension from their foundation (i.e., governance for law, knowledge for science, and values for human) to their ultimate goal (i.e., effectiveness for law, conservation for science, and health & prosperity for human). We further propose that the pathway for solutions should involve the continuous cycling across all dimensions interfaces to improve evidence‐based law, integrate conservation and use of resources, and develop holistic governance tools across the science‐policy interface; communicate science, increase community curiosity, and influence attitudes across the socioecological interface; and build political will, strengthen public support, and enhance industry backing at the sociopolitico‐legal interface (as represented in the outer cycle of Figure 2). Additionally, exploring how scientific advisory bodies contribute to global environmental law regimes could provide greater insights into how mismatches can be addressed. For example, nongovernmental organizations, such as the International Union for Conservation of Nature (IUCN), provide a critical service in species conservation (iucnredlist.org), but most of the existing conventions and global treaties, such as CMS and CITES, have their own scientific committees. Although common, this structure leads to siloes of information that may result in undesirable outcomes (Havice et al., 2018). Given the global endorsement of the IUCN Red List, it has been suggested that it could be more directly used as a source of scientific information to trigger and support listing decisions under treaties such as CITES (Frank & Wilcove, 2019). Alternatively, a shared advisory group would encourage communication between committees and serve to better inform treaty secretariats and states. The Joint Group of Experts on the Scientific Aspects of Marine Environmental Protection (GESAMP), for example, plays a valuable role in providing consistent scientific information on marine environmental protection to 10 UN bodies and related treaty secretariats. Facilitating regime interactions through multitreaty collaborations could provide another pathway for discussions across committees and facilitate the sharing of relevant information leading to more holistic governance (Young, 2011). Indeed, the BBNJ Agreement includes specific objects and principles focused on coherence with other instruments, integration and international cooperation (e.g., articles 5, 7, and 8). Also, the ITLOS Advisory Opinion confirms that, at least for UNCLOS, it is to be interpreted in light of later agreements (Yiallourides & Deva., 2024). Formalized existing partnerships between CBD, CMS, and CITES could be extended beyond multilateral environmental agreements to include other relevant treaties, such as the new BBNJ Agreement (https://tinyurl.com/3vztz8kv) and agreements addressing pollution (e.g., International Convention for the Prevention of Pollution from Ships [MARPOL] and the new plastics treaty being negotiated by the international community; [UNEP/EA.5/Res.14, March 2022]). The Food and Agriculture Organisation and RFMOs could also be better integrated to improve management and lead to better‐informed decisions.

By adopting a treaty, state parties have signaled political will for conservation. Yet, misalignments between the implementation of commitments from state parties and the impacts of their industrial activities on marine megafauna should be better assessed. This could be done by overlaying the spatial extent of activities on mapped areas of importance for marine megafauna, together with the obligations from all treaty regimes that apply to different countries. Furthermore, community values associated with species and places, such as sacred sites or places associated with cultural values for totem species (Berkes, 2012; Verschuuren et al., 2010), could also be mapped and overlaid with the scientific and governance data. To match such disparate datasets (animal movement, threat intensity, treaty obligations, and community values), standardized scientific data and access sharing of noncontentious data are essential (e.g., Sequeira et al., 2021) and will be key to advancing the effectiveness of legal frameworks. The decision‐making criteria used for species listings under existing global regimes could also be better defined to specifically address the impacts of human activities and species’ functional traits and associated human goods and services.

It is clear that for public support to be garnered legal frameworks must align with sociocultural values that are themselves shaped by worldviews and power relations in societies (Pascual et al., 2023). Some marine megafauna, such as whales, are charismatic species that capture the imagination of the public (Walpole & Leader‐Williams, 2002) and have been the force behind a shift from extractive (fishing) to nonextractive (tourism) uses (Mazzoldi et al., 2019). In some cases, if successful, a change in sociocultural values can be better harnessed to influence political will and bolster industry support (as happened for whaling). In other cases, a shift in cultural values will need to outweigh traditional economic concerns. Where jobs and livelihoods depend on industries and activities that have negative impacts on marine megafauna, these shifts can have unequal impacts on the original stakeholders. Therefore, alternative livelihoods and industries, economic opportunities, and substitute goods and services must be identified and explored. This is likely to need a re‐evaluation of marine megafauna to include, in addition to their ecological value, a clear attribution of their economic value (including use and nonuse values) (Sequeira et al., 2024). Other marine megafauna (such as sharks) are less charismatic and do not garner the same public support as whales. Yet, their ecological value is significant, and with improved science communication, public perceptions can change (Mazzoldi et al., 2019). In this sense, the full spectrum of values of nature, and the role they play in decision‐making, must be fully explored (Pascual et al., 2023).

Ultimately, it is governments that adopt and implement laws, and they are influenced by people, including individual voters, communities and groups, industries, and corporations. For scientific knowledge to be more effectively embedded in law, political will must be garnered and that in turn involves harnessing public support and overcoming industry lobbying resistant to change if industrial activities are to be curtailed. For industry, more stringent environmental regulations can increase compliance costs, yet they can also have a net positive effect on competitiveness by incentivizing cost‐cutting and greater efficiency (Dechezlêpretre & Sato, 2017). In some cases, curtailment might not be necessary. For example, the shipping industry faced a shift 30 years ago, when the images of oil‐covered seabirds and marine mammals shocked the world. This led, in part, to political will to adopt strict vessel construction regulations (through the International Maritime Organisation and MARPOL), whereby double hulls were required to prevent oil spills. Shipping was not curtailed, and thousands of marine megafauna species benefitted.

## CONCLUSION

Existing scholarship on mismatches affecting biodiversity conservation has been strongly focused on the 2‐dimensional science‐law interface. We argue that the mismatches are much more complex and occur as a multidimensional array across science, law, and human dimensions and in the short, medium, and long term. Akin to the solution of a Rubik's Cube, strict alignment across all the 6 dimensions is needed for successful biodiversity conservation. Focusing on marine megafauna, we demonstrated the interplay of existing mismatches that lie between the science, law, and human dimensions with some examples on how these may vary across the 3 temporal scales, and we shed light on ways forward to address the wicked conservation problems we now face. Importantly, we showed that the often less regarded socioecological and sociolegal aspects are key to conservation success. Continuous cycling across the interfaces of all dimensions is crucial to tease out where mismatches occur and to maintain alignments across temporal scales. This is particularly important because the medium and long term, respectively, become short and medium term (and so on). As the story of whaling and the rebound of whale numbers shows, much can be achieved when science, law, and society all swim in the same direction and all faces of the Rubik's cube align.
